# Acute Promyelocytic Leukemia and Severe Differentiation Syndrome in Pregnancy—A Management Challenge

**DOI:** 10.3390/life13051141

**Published:** 2023-05-08

**Authors:** Gabriela Droc, Maria Camelia Stancioaica, Corina Gabriela Soare, Mihai-Gabriel Stefan, Daiana Ingustu, Cristina Martac, Daniel Coriu, Sebastian Isac, Nicolae Suciu, Stefan Andrei

**Affiliations:** 1 Department of Anesthesiology and Intensive Care I, ‘Fundeni’ Clinical Institute, 022328 Bucharest, Romania; gabriela.droc@umfcd.ro (G.D.); ingustu.daiana@gmail.com (D.I.); christtina_martac@yahoo.com (C.M.);; 2 Department of Anesthesiology and Intensive Care I, Carol Davila University of Medicine and Pharmacy, 020021 Bucharest, Romania; 3 Department of Haematology, ‘Fundeni’ Clinical Institute, 022328 Bucharest, Romania; stancioaicamariacamelia@yahoo.com (M.C.S.); daniel.coriu@umfcd.ro (D.C.); 4 Department of Anaesthesiology, Cork University Hospital, College Rd. Wilton, T12 DC4A Cork, Ireland; soare.corinaa@gmail.com; 5 Department of Anaesthesiology and Intensive Care Medicine II, “Prof CC Iliescu” Emergency Institute for Cardiovascular Diseases, 022322 Bucharest, Romania; mihai.stefan@drd.umfcd.ro; 6 Department of Haematology, Carol Davila University of Medicine and Pharmacy, 020021 Bucharest, Romania; 7 Department of Physiology, Faculty of Medicine, Carol Davila University of Medicine and Pharmacy, 020021 Bucharest, Romania; 8 Department of Obstetrics and Gynecology, National Institute Mothers and Children Health “Alessandrescu-Rusescu”, 011061 Bucharest, Romania; nicolae.suciu@umfcd.ro

**Keywords:** acute promyelocytic leukemia, pregnancy, organogenesis, spontaneous abortion, all-*trans* retinoic acid, idarubicin, differentiation syndrome

## Abstract

Acute promyelocytic leukemia (APL) is generated by the PML-RARA fusion gene. In patients suffering from APL, the early diagnosis and treatment are essential in the successful management. We reported a case of a 27-year-old 17th-week pregnant patient diagnosed with APL. After an extensive hematological diagnostic panel, the acute promyelocytic leukemia was confirmed, and the patient received all-*trans* retinoic acid (ATRA), idarubicin (IDA), and dexamethasone, following national guidelines. Due to ATRA-related differentiation syndrome, the therapy was adjusted, and hydroxycarbamide was added with a good outcome. The patient was admitted to the ICU secondary to hypoxemic respiratory failure on the 2nd day after hospital admission. Our patient received an individualized drug combination, adjusted by the clinical response. Furthermore, the drugs used in APL treatment are all teratogenic. Despite various major complications, including severe acute respiratory distress syndrome (ARDS), which needed mechanical ventilation; ICU-acquired myopathy; and spontaneous abortion, the patient had a good outcome and was transferred from the ICU after a total stay of 40 days. APL during pregnancy is a rare entity of intermediate-risk APL. Our study emphasized the need for individualized therapy in a rare case of a pregnant woman diagnosed with a potentially fatal hematologic disease.

## 1. Introduction

Acute myeloid leukemia (AML) is a type of hematologic malignancy with a high prevalence mainly in elderly patients. However, there are many cases of the young population, as well [1]. The diagnosis of AML must be supported by morphological, immunophenotypic, cytogenetic, and molecular tests. Acute promyelocytic leukemia (APL) is a subtype of acute myeloid leukemia (AML). APL is characterized by a balanced translocation t(15;17) (q24;q21) that generates the PML-RARA fusion gene, conferring sensitivity to treatment with anthracycline-based chemotherapy and differentiating agents, such as all-trans-retinoic acid (ATRA), with a reported curative rate of approximately 90% [2,3,4]. The actual standard therapy for low-risk APL consists of the association of ATRA and arsenic trioxide (ATO) [5]. Other agents, such as idarubicin (IDA) and hydroxycarbamide, could be added in accordance with national guidelines and illness progression [6].

In association with pregnancy, APL can lead to severe complications, including maternal and/or fetal death [2,7]. The literature on APL and pregnancy and peripartum is limited, with only 96 cases having been reported to date [8]. The treatment of APL during pregnancy is challenging, and an individualized approach should be applied based on local expertise and national guidelines. Due to teratogenicity, ATRA should be avoided in the first trimester, with consequent retinoid embryopathy [3]. The decision-making process in this difficult clinical scenario is complex and focuses on the choice of the most suitable therapeutic agents given the increased maternal and fetal risks, gestational age, and ethical considerations [8].

Differentiation syndrome (DS), previously known as retinoic acid syndrome, is the main life-threatening complication associated with induction therapy, using differentiating agents in patients with APL, in the presence of leukemic blasts [9]. The syndrome is characterized by fever, acute respiratory distress syndrome (ARDS), acute kidney injury (AKI), vascular capillary leak syndrome, and distributive shock, which requires supportive therapy [9,10].

We describe the case of a pregnant patient diagnosed with APL in the second trimester of pregnancy who developed severe differentiation syndrome (secondary to the administration of ATRA) and shock, requiring prolonged ICU management.

## 2. Case Presentation

A 27-year-old obese (BMI of 37.83 kg/m^2^) pregnant patient (G2P1) without any positive history of genetic or hematologic malignancies was diagnosed with APL during the 17th week of gestation.

The initial symptoms were non-specific, consisting of fever (38.5 °C) nausea, vomiting, and ongoing fatigue lasting for one week before her first presentation. The clinical picture upon admission was unremarkable, with no apparent obstetric complications.

The full blood count revealed pancytopenia with white blood cell count (WBC) of 2.91 × 10^3^/μL (3.90–10.00), hemoglobin level (Hb) of 8.5 g/dL (11.2–17.5), platelet count (PLT) of 15 /μL (150–450), INR 1.5 (0.8–1.2), fibrinogen of 151 mg/dL (200–400), CRP of 278 mg/L (0–3), and LDH of 600 mg/dL (208–378).

Differential diagnoses were maternal sepsis, pregnancy-induced thrombocytopenia, or hematologic malignancy. After a full sepsis workup, broad-spectrum antibiotics were initiated with piperacillin/tazobactam.

The extended hematologic panel on admission pointed out the diagnostic criteria for APL: peripheral blood film showed 92% promyelocytes with bi-lobed nuclei and azure granules and some with cytoplasmic Auer rods. The bone marrow aspirates identified over 95% promyelocytic blasts, with MPO + 100%. Flow cytometry and a large population of blasts comprising 62% with an APL immunophenotype: CD34− CD117+low, CD13+, CD33+, CD64+, CD123+ CD38+ CD15+ low, CD14−, HLA-DR−, CD7−, and CD56−.

The fluorescence in situ hybridization (FISH) for the PML/RARA fusion showed that 84% of cells had the t(15;17)(q22;q21) translocation, resulting in PML/RARA gene fusion. The chromosome analysis showed that 10/21 cells had 46, XX, t(15;17)(q22;q21). The molecular biology for the PML-RARA bcr2 gene fusion was also positive. Low/intermediate-risk acute promyelocytic leukemia was diagnosed. Following an extensive discussion with the patient and after giving special consideration to the treatment side effects, a decision was made to initiate the ATRA 45 mg/m^2^ orally BD, IDA 25 mg IV every 48 h (2 doses in total), and dexamethasone (2.5 mg/m^2^ IV BD).

DS developed 24 h after the beginning of ATRA, associating progressive peripheral edema and dyspnea with polypnea and severe coagulopathy. Based on the clinical status degradation and CT scan, ATRA was interrupted on day 2, and the dose of dexamethasone was increased to 10 mg IV BD. Antibiotic therapy was escalated to linezolid 600 mg IV BD and meropenem 1 g IV TDS, while piperacillin/tazobactam was discontinued.

**Figure 1:**
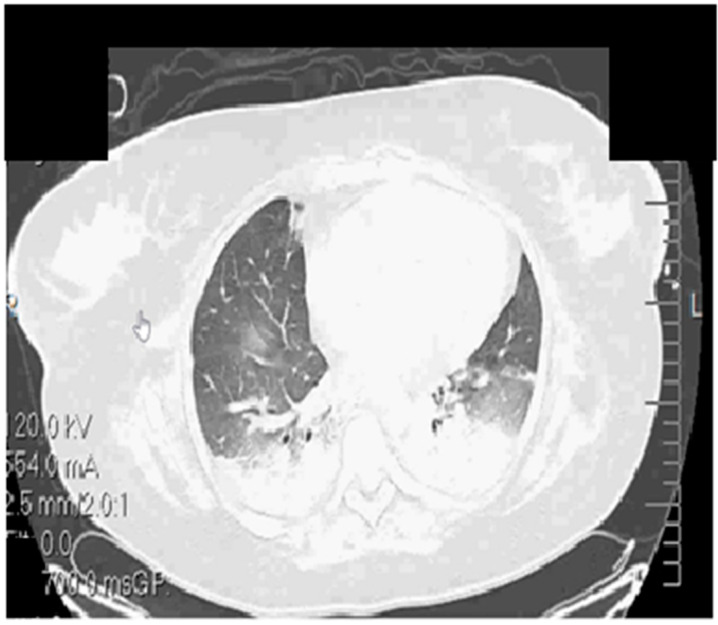
Native thorax CT at admission.

The initial CT Thorax scan identified multiple bilateral pulmonary infiltrates (Figure 1).

The patient was admitted to the ICU on the 2nd day of treatment for acute hypoxemic respiratory failure and fluid overload unresponsive to diuretic therapy. The sequential organ failure assessment (SOFA) score upon admission to ICU was 8, with an associated mortality rate of 60%.

Upon ICU admission, the patient had a severe respiratory compromise, dyspnea with tachypnea, bilateral crackles on auscultation, and hypoxemia. The Sp0_2_ was 86% despite oxygen administration at a flow of 14 L/min via a facemask. The neurological status was unaltered, and the patient was afebrile and hemodynamically stable. Subsequently, the patient’s status progressively worsened, developing ARDS, hemodynamic instability, acute kidney injury, and persistent coagulopathy in the context of a very severe DS post-ATRA.

The biochemistry profile revealed the following: Na^+^ of 137 mmol/L (132–146), K^+^ of 3.15 mmol/L (3.5–5.5), Ca^++^ of 1.21 mmol/L (8.7–10.4), Cl^−^ of 104 mmol/L (99–109), blood glucose of 131 mg/dL (74–106), CRP 287 (0–3), ALT of 65 U/L (0–49), AST of 83 U/L (0–34), and total bilirubin of 2.35 mg/dL (0.1–1.2). The results of the repeated FBC and coagulation test were as follows: WBC, 56.76 × 10^3^/μL (3.90–10.00); Hb, 7.40 g/dL (11.2–17.5); PLT of 5/μL (150–450); fibrinogen of 66.5 mg/dL (200–400); and INR of 2.01 (0.7–1.2).

**Figure 2:**
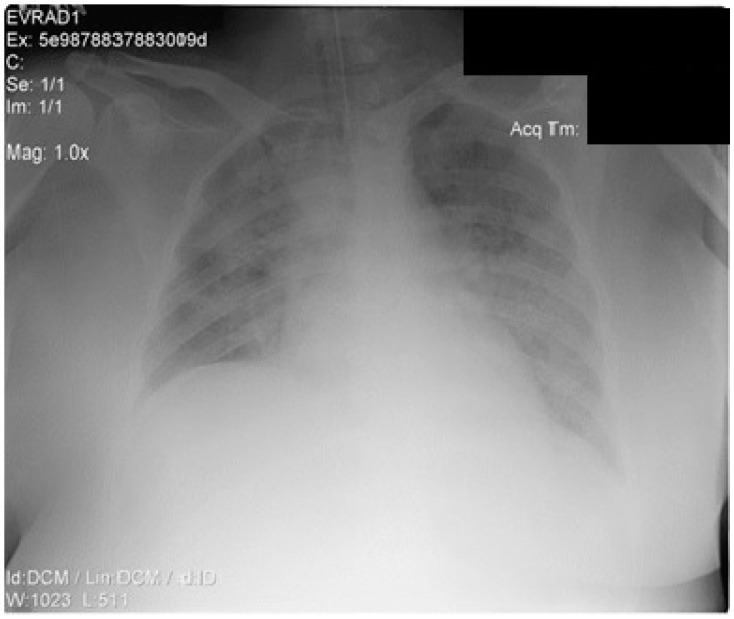
Thorax X-ray after intubation (anteroposterior view).

Despite the administration of supplemental oxygen, followed by a high-flow nasal cannula, the respiratory function declined rapidly. The chest X-ray is shown in Figure 2. The patient required intubation and protective mechanical ventilation on day 1, soon after being admitted to the ICU for severe ARDS (P/F < 150), along with increasing oxygen requirements (FiO_2_ up to 70%), higher PEEP levels, continuous sedation, and neuromuscular blockade. Repeated PCR SARS-COV-2 tests were performed upon admission, day 4, day 9, day 14, and day 36, all with negative results.

Distributive shock required the administration of high-dose vasopressor agents (norepinephrine up to 0.4 μg/kg/min) to maintain a mean systolic blood pressure >65 mmHg from day 1 in the ICU. The vasopressor requirement decreased during the first week and was stopped on ICU day 7. Serum lactate on admission to ICU was 3.67 mmol/L reaching a peak of 6.5 mmol/L in 24 h and normalizing 6 days later.

In the presence of anuria, fluid overload, and metabolic acidosis, acute continuous venovenous hemodiafiltration was initiated on day 2 in ICU, for a total of 4 days. Spontaneous termination of pregnancy occurred 36 h after the administration of the second dose of IDA, complicated by postpartum hemorrhage, secondary to associated coagulopathy. The obstetrical assessment was needed for the next following 3 days, and repeated blood transfusions were required for recurrent episodes of post-procedural bleeding. No further obstetrical invasive procedures or other pharmacologic treatments were indicated.

Blood cultures (one performed on hospital admission and the second on day 1 in the ICU) were positive for methicillin-resistant Staphylococcus aureus (MRSA). Epidermal excoriations of the abdominal wall identified initially upon clinical examination were considered a potential entry point. The transesophageal echocardiography showed no signs of bacterial endocarditis. The first negative blood culture was recorded on day 6 in the ICU.

Regarding the hematological status and considering the elevated WBC on the 3rd day after admission, hydroxycarbamide at 3 g daily for a total of 2 days was initiated. The dose of dexamethasone was increased up to 16 mg BD and continued for 7 days. The ATRA was reintroduced on the 5th day after admission, considering the favorable short-term respiratory evolution and the early disruption of the treatment. The dose was, however, reduced to 25 mg/m^2^, orally BID.

**Figure 3:**
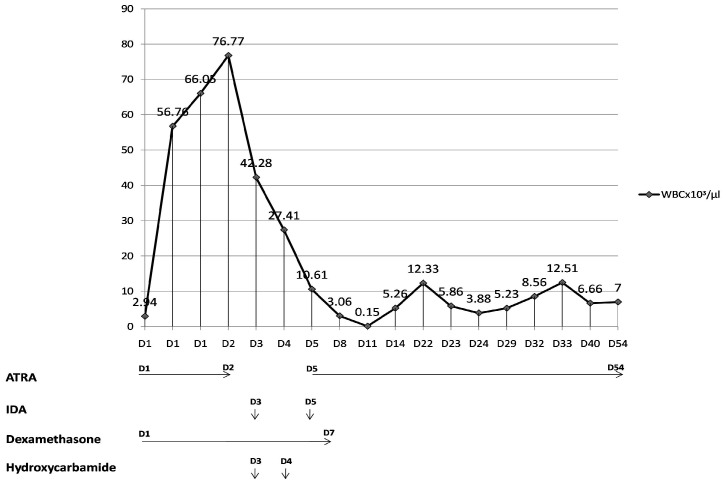
White blood cell dynamics during hospital stay and the etiological treatment. D—day of hospital stay; ATRA—all-*trans* retinoic acid; IDA—idarubicin.

The repeated blood workup revealed pancytopenia on the 8th day after ICU admission: WBC 0.2 × 10^3^/μL (3.90–10.00), Hb 6.7 g/dL (11.2–17.5), PLT 15,000 × 10^3^/μL (150–450), D-Dimers 18,900 ng/dL (0–500), fibrinogen 105 mg/dL (200–400), and INR 1.48 (0.7–1.2) in keeping with DIC. Changes suggestive of acute kidney injury: creatinine 1.13 mg/dL, urea 246 mg/dL, and acute liver injury; total bilirubin 3 mg/dL (0.1–1.2), AST 227 UI/L (0–34), and ALT 183 UI/L (0–49) were also present. Considering the IDA-associated neutropenia and the current clinical picture, G-CSF (granulocyte-colony-stimulating factor) therapy was initiated on the 8th day after admission and continued for a total of 4 days. The gradual improvement in blood count and coagulation profile was noted after 25 days in ICU (Figure 3).

The overall clinical picture improved gradually, allowing sedation cessation on day 26. An initial spontaneous breathing trial, followed by extubation, was attempted on day 30 in the ICU; however, secondary to ICU-acquired myopathy, the patient required reintubation for 5 more days and was subsequently weaned off mechanical ventilation to HFNC on day 35. On day 40, the patient was transferred to the hematology ward. After 54 days of hospital stay, the patient was discharged with functional status of 3/5 and required continuous physiotherapy at home. The hematological follow-up showed complete morphological remission. The long-term etiological treatment consisted of three consolidation cycles according to the AIDA2000 protocol, followed by 2 years of maintenance treatment [11]. Long-term disease follow-up did not show any PML-RARA fusion gene.

## 3. Discussion

We presented the challenging yet successful management plan of a case of severe post-ATRA DS in a pregnant patient, complicated by spontaneous termination of pregnancy and multiple organ failure requiring prolonged ICU stay. The presence of MRSA bacteremia in this instance could have been a marker of sepsis, partially explaining the rapid initial deterioration or just an incidental finding.

Our patient developed multiple organ dysfunctions, with respiratory, cardiovascular, neurological, renal, hematological, and hepatic involvement. Despite the guarded prognosis, prompt ICU admission and the multidisciplinary team approach positively impacted the overall patient outcome. Some but not all reviewed cases reported mild differentiation syndrome [8,12,13]. In Santolaria’s review, the causes of death were reported in 7 patients (out of a total of 10 deaths): 3 intracranial hemorrhages, 3 cases of multiorgan failure (2 caused by severe differentiation syndrome), and 1 caused by infection [8]. In our case, we identified the presence of multiorgan failure and associated infection with a fulminant worsening of the clinical picture.

The pathogenesis of DS is complex; ATRA administration leads to releasing a variety of cytokines by differentiating blast cells, changing the adhesive properties of blast cells and conducting a systemic inflammatory response syndrome [14]. The combination of a systemic inflammatory state with increased vascular permeability and endothelial damage results in hypotension and organ hypoperfusion, which ultimately lead to multi-organ dysfunction [14]. Moreover, considering the pregnancy-related unpredictability of the immune response to a hematological disease and the dynamic of the WBC count in the first 24 h, we decided to introduce dexamethasone as the primary prophylaxis for DS.

Our patient was seventeen weeks pregnant, and the termination occurred seven days after initiating ATRA treatment and after the second dose of IDA. Administration of ATRA or chemotherapy in the first trimester of pregnancy is associated with an increased risk of fetal malformations and spontaneous abortion, whereas administration during the second and third trimesters is associated with a better fetal outcome [15]. Since no consensus exists with regard to pharmacological termination of a pregnancy in the second trimester for patients with APL and ATRA-related DS and considering the increased risk of bleeding at admission to the hospital, we decided to inform the patient about the risk of spontaneous abortion and consider an obstetrical supportive therapy. Moreover, according to Santolaria et al., 70% of the pregnancies in the second trimester had a good fetal outcome [8]. Conversely, our patient experienced a spontaneous abortion that could have been caused by the presence of DS and disseminated intravascular coagulopathy or could have been the consequence of hypoxemia secondary to severe anemia, ARDS, and hemodynamic instability. Although experience with ATRA in pregnant patients is limited, retinoids are known to be potent teratogens [9]. Increased rates of spontaneous termination of pregnancy and major fetal abnormalities have been reported [9,16]. This is most likely the underlying mechanism that led to the clinical picture encountered in our patient.

Out of the 88 followed-up patients, 78 (89%) achieved complete remission [8]. This was also the case with our patient, despite a prolonged hospitalization and ICU stay.

## 4. Conclusions

In conclusion, we presented a case of a young patient diagnosed with low/intermediate APL in the second pregnancy trimester, emphasizing the need of prioritizing the mother’s life in a context of a severe hematologic malignancy. Finally, our case pointed out the need for an individualized multidisciplinary approach in ICU settings.

## Data Availability

No new data were created or analyzed in this study. Data sharing is not applicable to this article.
